# The impact of enriching heat-stressed rabbit diets with flaxseed oil with/ without allicin, lycopene, or Punicalagin on antioxidative status, physiological response and meat omega-3

**DOI:** 10.1186/s12917-025-04615-0

**Published:** 2025-03-20

**Authors:** Yassmine M. El-Gindy

**Affiliations:** Department of Animal and Fish Production, Faculty of Agriculture (Saba Basha), Alexandria University, P. O. Box 21531, Alexandria, Egypt

**Keywords:** Heat stress, Flaxseed oil, Allicin, Lycopene, Punicalagin, Meat ω3 and ω 6, Antioxidants

## Abstract

To improve the health and lifespan of customers, modern nutritionists have focused on improving meat quality and nutritional value. To enhance the unsaturation lipids of rabbit meat, different oil sources used in rabbit diets. Flaxseed oil (FSO) is one way to raise the polyunsaturated omega-3 fatty acid (ω3) in animal meats. On the other hand, FSO can cause high rates of oxidation in rabbit meat under heat stress. Therefore, the use of natural antioxidants may be a good strategy to produce functional meat under stress. The study investigated the beneficial impact of enriching heat stressed rabbit diets with FSO supplemented with/without allicin (ALC), lycopene (LCO) or punicalagin (PCA) during the last 3 weeks before slaughter at a ban temperature ranging from 30 to 35 C, humidity 70 to 82% and temperature humidity index of 27.9 to 31.2 on growth performance, antioxidative status, physiological response, liver and kidney function and ω3 & ω6. In total, 120 male V-line growing rabbits (5 weeks old) were divided into 5 groups, 24 rabbits each. CON rabbits fed a standard diet without supplementation, FO rabbits fed a diet with 1.5% FSO, FOA rabbits fed a diet with 1.5% FSO and 100 mg / kg of ALC diet, FOL rabbits fed a diet with 1.5% FSO with 100 mg LCO / kg of diet, FOP rabbits fed a diet with 1.5% FSO with 100 mg PCA / kg of diet. All treatments with flaxseed oil supplemented with antioxidants significantly elevated ω3 content and ratio between ω3/ω6 of rabbit meat (*P* ≤ 0.01) while concomitantly reducing total cholesterol without any significant change in physiological response (rectum temperature and respiration rate). Furthermore, these treatments markedly improved antioxidant status, as evidenced by increased total antioxidant capacity and decreased lipid peroxidation. Additionally, serum immunoglobulin G (IgG) significantly (*P* ≤ 0.01) elevated in FOA, FOL and FOP rabbits compared to the CON group. Supplementation with ALC, LCO and PCA could be recommended to restrain the results of oxidative stress results of enriched diets with ω3 and heat stress to provide healthier and functional rabbit meat (rich in ω3).

## Introduction

The main aim of animal scientists, researchers and nutritionists is to boost rabbit meat of polyunsaturated ω3in rabbit meats with improvement in farm animal productivity [1]. Monogastric animals, such as rabbits, can convert dietary fatty acids into fatty tissue and intramuscular fat, allowing the fatty acid profile of rabbit meat to be changed using polyunsaturated dietary fat [2, 3]. Functional foods consider a possible way to reduce public health costs by improving the health situation by reducing the risk of disease [4]. The global study has focused on expanding the ω3in animal origin products by enriching diets with oils or raw materials that are high in ω3 PUFA such as FSO [4, 5]. Flax seed oil is rich in polyunsaturated fatty acids (ω3), particularly α-linolenic acid, comprising at least 50% of its total fatty acid content [1].

Several studies have confirmed the beneficial effects of natural antioxidants, including their ability to boost the immune system, mitigate lipid peroxidation, and provide potent antioxidant protection. Flaxseed (*Linum usitatissimum*), cultivated primarily for oil and animal feed, is a valuable source of PUFA. These essential fatty acids include palmitic acid, stearic acid, oleic acid, linoleic acid, and α-linolenic acid [1]., which cannot be produce by animal and human bodies. Furthermore, FSO promotes health by helping prevent and treat coronary heart disease, arthritis, and high blood pressure [6]. Feeding a diet enriched ω3 PUFA during the last two to three weeks of the fattening period can significantly boost the ω3 PUFA content of the meat, while also lowering overall production costs compared to longer-term supplementation [7].

On the other hand, global warming is a great challenge for humans and rabbits, which already exposed to heat stress when temperatures rise above 18 °C, especially under conditions of high humidity. Heat stress considers the most environmental stressors, which have the most harmful consequences on animal models in physiological, biochemical, metabolic, and cellular responses. Therefore, the feed could be more susceptible to oxidation and tended to decrease the intake of feed and fiber digestibility, especially in conditions of high environmental temperature. Heat stress motivates ROS production [8]. The elevation of PUFA content in rabbit meat, induced by dietary supplementation with FSO, can result in accelerated oxidation and reduced shelf life [9]; this means that antioxidant supplementation is required.

Antioxidants could be a good strategy to restrict or prevent the development of oxidative stress and protect PUFA of FSO from damage by peroxidation by improving the taste, texture and nutritional value of rabbit meat, while extending its storage life [5]. Using antioxidants such as ALC, LCO, or PCA could mitigate oxidative stress and enhance growth performance. ALC, a potent organosulfur compound extracted from garlic, a member of the *Alliaceae* family [10]. Allicin is characterized by its strong, pungent odor and a variety of beneficial properties, including antibacterial, antifungal, anti-inflammatory and antioxidant effects [11]. Lycopene (a natural red tomato pigment and one of the strongest antioxidants among carotenoids [12]), potentially plays a significant role in the body’s antioxidant defense system [13], by neutralizing harmful ROS. This protective action could be particularly advantageous for rabbits under stress, accelerated growth, high reproductive rates, and the intensive metabolic demands of modern animal farming. Punicalagin extracted from pomegranate (*Punica granatum*), it is very rich in polyphenols and flavonoids [14]. Punicalagin, the primary bioactive compound in pomegranate peel, exhibits antioxidant and anti-inflammatory effects [15]., antimicrobial [16], tissue protective effects [17], and anticancer properties [18].

In view of the protective role of ALC, LCO, and PCA against the different stressors mentioned above, it has been hypothesized that ALC, LCO and PCA could be an ameliorative strategy to produce meat rich in ω3 under stress of summer heat and FSO diets. Therefore, the primary purpose of this research was to determine the beneficial impact of feeding a diet rich in FSO with/ without ALC, LCO or PCA on antioxidative status, physiological response, meat fatty acid profile of ω3 and ω6 in growing heat stressed rabbit. Although previous studies have explored the individual effects of FSO and natural antioxidants, their combined impact on heat-stressed rabbits remains unexplored. This pioneering study investigates the possible synergistic effects of FSO and natural dietary antioxidants on immune function, physiological response, and meat quality (omega-3 and omega-6 fatty acid composition) over a short-period of 3 weeks before slaughter in heat-stressed rabbits.

## Materials and methods

### Ethics statement and study site

The experiment was performed at the Rabbit Laboratory, Faculty of Agriculture (Saba Basha), Alexandria University, Egypt (31°12’20.7108"N, 29°55’28.2936"E). The V-Line rabbits taken from a research farm of the Faculty of Agriculture of the University of Alexandria, Egypt. All experimental procedures involving rabbits conducted in accordance with ethical guidelines and approved by the University Institutional Animal Care and Use Committee (Approval No. 14/20/11/1//3/14). The experiment conducted during the summer season of 2022 (August to September) in accordance with the relevant guidelines and regulations for animal research, including the recommendations of the ARRIVE Guidelines.

### Animals, experiment schedule, and diet

Hundred and twenty V line male rabbits, 5 weeks old (676.88 ± 26.91 g) were exposed to heat stress during the summer season (begin of July - mid-August 2022). Then after that, the growing rabbits were adapted to the experiment diets for 7 days, then the experiment lasted 21 days before slaughter (mid-August - mid-September 2022). The rabbits were divided into five random groups of 24 rabbits each, with 8 replicates per group (3 rabbits each). All rabbits were accommodated in standard cages, 3 rabbits per cage. The rabbit cages were wire floor batteries of 50 × 50 × 40 cm in the same hygienic condition and in a natural ventilated block building with exhaust fans. Five different pelleted diets were formulated for CON rabbits– fed a standard diet without supplementation, FO rabbits fed a pelleted diet with 1.5% FSO (according to [19]), FOA rabbit fed a pelleted diet with 1.5% FSO and 100 mg/kg diet ALC purchased from Double Ok Life Co., Ltd-Fujian China, mainland, FOL - fed a pelleted diet with 1.5% FOS and 100 mg / kg diet LCO purchased from Roche, Levent-Istanbul, the recommendation doses of allicin and lycopene are according to [20], FOP rabbits fed a pelleted diet with 1.5% FSO plus 100 mg/kg diet PCA obtained from MedChem Express (USA), the dose is according to [21]. Experimental diets (Table 1) and fresh water offered to rabbits *ad libitum.* Chemical analysis of all treatment diets (protein, fiber, and digestive energy) calculated.

**Table 1:** Composition and chemical analyses of control and the basal flaxseed oil experimental diets

Ingredients	Control	Flaxseed oil 1%
Clover hay	28.00	28.00
Yellow corn	16.90	16.90
Wheat bran	11.50	11.50
Barley grain	17.30	17.30
Soybean meal (44%)	20.00	20.00
Molasses	3.00	3.00
Linseed oil	------	1.50
Sunflower oil	1.50	------
Limestone	1.00	1.00
Nacl	0.30	0.30
Dl- Methionine	0.10	0.10
L-Lysine	0.10	0.10
Vit, and min. mix.^1^	0.30	0.30
**Calculated value (%)**		
Crude Protein	17.19	
Crude Fiber	12.64	
Ash	7.60	
DE^2^ (kcal/ kg DM)	2743.50	

^1^Vit+Min mixture provides per kilogram contains: Vit A 6000 IU; Vit D_3_ 450 IU; Vit E 40 mg; Vit K_3_ 1 mg; Vit B_1_ 1 mg; Vit B_2_ 3 mg; Vit B_3_ 180 mg; Vit B_6_ 39 mg; Vit B_12_ 2.5 mg; Pantothenic acid 10 mg; biotin 10 mg; folic acid 2.5 mg; choline chloride 1200 mg; Manganese 15 mg; Zinc 35 mg; Iron 38 mg; Copper 5 mg; Selenium 0.1 mg; Iodine 0.2 mg; Selenium 0.05 mg. ^2^Digestible energy (kcal/kg DM) calculated according to Fekete and Gippert (1986) using the following equation: DE (kcal/ kg DM) = 4253 − 32.6 (CF %) -144.4 (total ash)

### Microenvironment condition

During the study period, air temperature and humidity % within the rabbitry measured at 08:00 AM and 3:00 PM via a hydro-thermometer. Furthermore, the temperature-humidity index (THI) was determined and classified on its implications for rabbit thermoregulation by the equation. THI = db ° C [(0.31 0.31 (RH)) (db ° C 14.4)], defined by where db ° C = the temperature of the dry bulb, and RH = the relative humidity / 100 [22]. The THI values were categorized according to [23] as follows:

Less than 27.80 = absence of heat stress; from 27.80 to 28.90 = moderate heat stress; from 29.00 to 30.00 = severe heat stress; more than 30.00 = very severe heat stress. The rabbits maintained under 16 h of light followed by 8 h of darkness each day. Thermoregulatory indices (rectal temperature (RT) and respiration rate (RR)) measured in rabbits. RT measured by inserting a clinical thermometer 3 to 5 cm into the rectum for 2 min, angled toward the rectum wall. RR was determined by visually counting movements per minute without disturbing the rabbit using a stopwatch.

### Performance traits

Initial and final body weight (FBW) and feed intake (FI) recorded. Also, the body weight gain (BWG) = initial BW- final BW, and feed conversion ratio (FI/gain) evaluated.

### Blood sample and biochemical analysis

At the end of the experimental period and before slaughter, blood samples were withdrawn by syringe from Marginal ear vein and collected in vacuum test tubes and the samples coagulate at room temperature then centrifuge for 20 min at 700 g to separate serum and kept at -20 ° C for biochemical analysis to measure the levels of total protein, albumin, total lipids, cholesterol, triglycerides, high-density lipoprotein, v-LDL, creatinine, urea, GOT, GPT, TAC, and MDA. Biochemical analyses measured colorimetrically by Bio-diagnostic, Cairo, Egypt, following the procedure outlined by the manufacturer. Globulin concentration calculated by determining the difference between total protein and albumin levels. This is because fibrinogen, another protein component, usually has a negligible impact on the overall protein profile [24]. Low-density lipoprotein cholesterol (LDL) was calculated by [25] Formula: LDL = TC-HDL-TG/5.

### The muscle fat acids analysis (ω3 and ω6)

Following the completion of the experiment, six rabbits per each group at 15 weeks old slaughtered by cutting the jugular vein, immediately after complete bleeding, tissue samples of the hind leg muscle obtained. The hind leg meat was stored at -20 ° C. Muscle lipids were extracted from hind leg muscle tissue of rabbit and measuring ω3 and ω6 by using gas chromatography (GLC) via thin layer chromatography for separation of lipid classes by [26]. Muscle cholesterol was measured via Richmond [27] method, using CHOD-PAP Cholesterol CHOD-PAP Kits manufactured by Human, Germany.

### Immune response

Rabbit serum samples analyzed for IgG levels using a quantitative ELISA assay (ELISA Quantification Kit, Bethyl Laboratories, UK).

### Statistical analysis

IBM SPSS Statistics 20 used to perform a one-way ANOVA on the experimental data. Single rabbits served as experimental units for analytical determinations, while groups of three rabbits considered experimental units for performance parameter assessments. One-way analysis of variance (ANOVA) performed using IBM SPSS Statistics 20 to analyze the experimental data. Individual rabbits served as experimental units for analytical determinations, while groups of three rabbits considered experimental units for performance parameter assessments.

To identify significant differences among group means, a one-way ANOVA performed. When the overall F-test was significant (*p* < 0.05), pairwise comparisons performed using Tukey’s HSD test.

The statistical model used for one-way ANOVA is as follows:

$$\:YiK\hspace{0.17em}=\hspace{0.17em}\hspace{0.17em}+\hspace{0.17em}Xi\hspace{0.17em}+\hspace{0.17em}eik$$

where:

YiK: Response variable; µ: Overall mean; Xi: Fixed effect; eik: Random error term. Data visualisation and statistical analyses performed with the aid of SigmaPlot version 14.0. “.

## Results

### Microenvironment condition and thermoregulatory indices

**Fig. 1:**
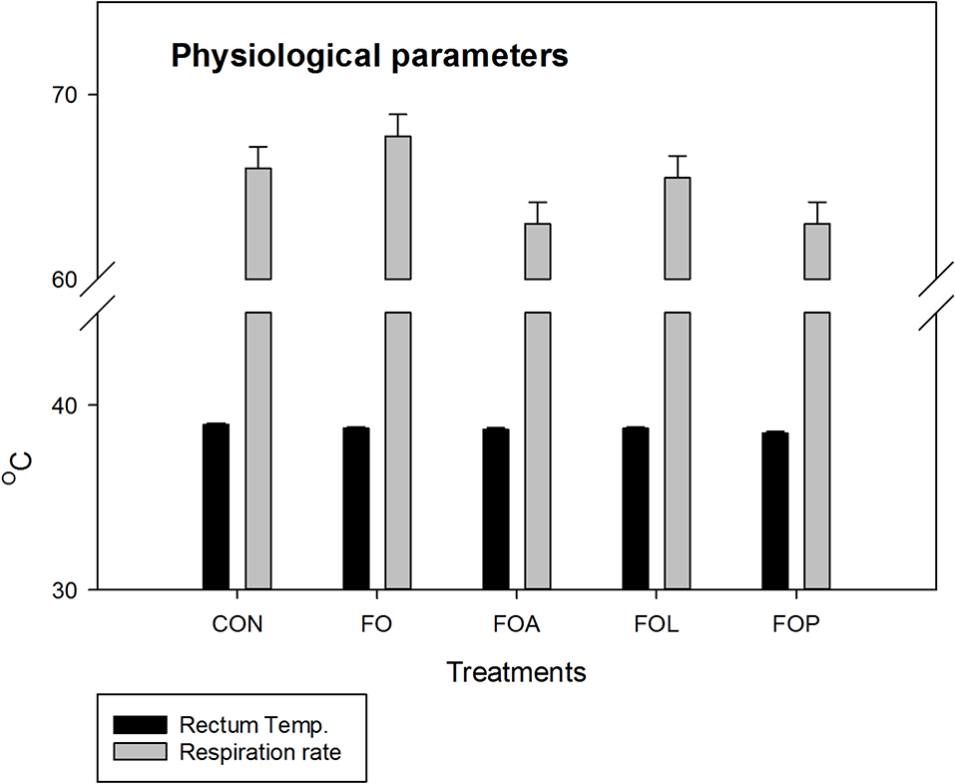
Effects of Flaxseed oil with or without allicin, lycopene and Punicalagin on physiological parameters: respiration rate (RR) and rectum temperature (RT) of heat stressed V-line growing rabbits. FO = Flaxseed oil; FOA = Flaxseed oil + Allicin; FOL = Flaxseed oil + Lycopene; FOP = Flaxseed oil + Punicalagin

Table 2 displays the weekly air temperature, RH, and THI throughout the 3 weeks of the experiment period. The temperature and relative humidity under the experimental conditions ranged from 29 to 33 °C & 75–85%, respectively. The THI values ranged from 27.87 to 32.02. Physiological parameters (RR and RT) at the third week of the experimental period shown in Fig. 1. The result showed that all experimental treatments do not affect the thermoregulatory indices of the growing rabbit under heat stress.

**Table 2:** Microclimate date of the experimental period (temperature, humidity, and temperature humidity index, THI)

Week	Temperature °C (Range)	Humidity % (Range)	THI (Temperature Humidity Index)
1^st^ Week	29–32	75–80	27.87–30.91
2^nd^ Week	29–33	77–83	27.96–32.02
3^rd^ Week	30–32	78–85	28.94–31.18

### Performance traits

The data in Table 3 indicate that the various treatments did not exert a significant influence (*p* = 0.532) on the feed conversion efficiency. However, a notable decrease in final body weight (*p* = 0.042), feed intake (*p* = 0.050) and BWG (*p* = 0.001) found in the FSO oil-treated group. In contrast, the inclusion of ALC, LCO, or PCA along with FSO resulted in an enhancement of these parameters. Among the experimental groups, the FSO + LCO combination produced the most favorable results in terms of FBW and BWG.

**Table 3:** Effects of flaxseed oil with or without allicin, lycopene and Punicalagin on growth performance

Treatment	control	Flaxseed oil				SEM*	*P* value
		alone	allicin	lycopene	Punicalagin		
Initial body weight g. at 12 wks.	1435.83	1422.92	1425.28	1431.67	1402.08	15.46	0.969
Final body weight g. at 15 wks.	1719.58^ab^	1674.17^b^	1833.96^ab^	1888.75^a^	1766.25^ab^	24.92	0.042
Body weight gain g.	283.75^bc^	251.25^c^	408.68^ab^	457.08^a^	364.17^ab^	18.18	0.001
Total Feed intake g.	1039.28^ab^	831.30^b^	1378.60^a^	1490.55^a^	1319.20^a^	83.24	0.050
Feed conversion ratio	3.76	3.31	3.36	3.30	3.74	0.11	0.532

Means in row with different superscript letters (a-c) are significantly different (*p* ≤ 0.05), *SEM = Stander Error Mean

### Biochemical analysis of blood

Dietary treatments FSO with/ without effects (ALC, LCO, or PCA) on blood biochemical analysis. Regarding the profile of blood lipids (Table 4), the levels of T. lipid, cholesterol, triglycerides, and v-LDL of FSO rabbits without antioxidants recorded the highest concentrations. While ALC succumbed to alternate (*p* = 0.023) and reduce the levels of blood triglyceride and v-LDL of FSO-growing rabbits. In addition, LCO significantly (*p* = 0.027) reduces the blood cholesterol level of rabbits growing FSO. Furthermore, each of ALC, LCO or PCA significantly decreased (*p* = 0.001) serum total lipid of the FSO group.

**Table 4:** Effects of flaxseed oil with or without allicin, lycopene and Punicalagin on blood lipid profile

Treatment	control	Flaxseed oil				SEM*	*P* value
		alone	allicin	lycopene	Punicalagin		
T. Lipid	401.00^ab^	473.50^a^	313.67^b^	324.00^b^	338.50^b^	15.60	0.001
Cholesterol	68.03^ab^	72.33^a^	60.77^ab^	57.13^b^	61.37^ab^	1.73	0.027
Triglyceride	74.17^ab^	78.67^a^	63.67^b^	66.00^ab^	69.70^ab^	1.69	0.023
HDL	25.37	31.17	31.90	26.87	27.83	1.00	0.175
LDL	27.83	25.43	16.13	17.07	19.59	1.75	0.125
v-LDL	14.83^ab^	15.73^a^	12.73^b^	13.20^ab^	13.94^ab^	0.34	0.023
HDL/LDL	1.24	1.79	2.39	1.70	1.46	0.19	0.394

Means in row with different superscript letters (a-b) are significantly different (*p* ≤ 0.05), *SEM = Stander Error Mean

**Fig. 2:**
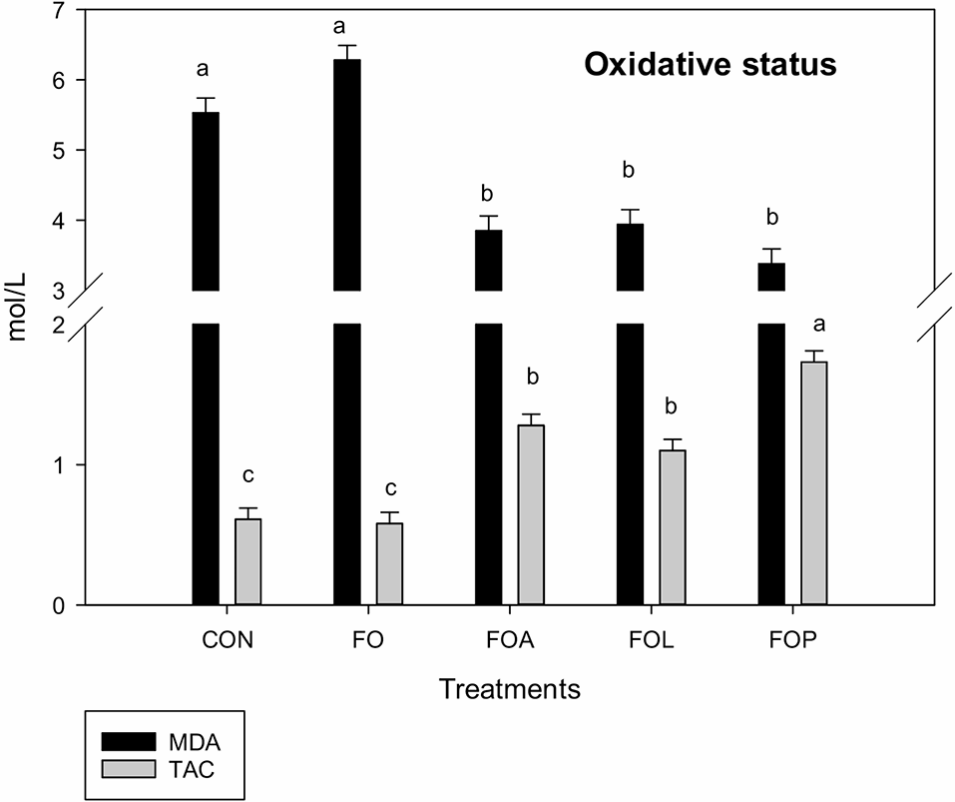
Effects of Flaxseed oil with or without allicin, lycopene and Punicalagin on oxidative status: malondialdehyde (MDA); total antioxidant capacity (TAC) of heat stressed V-line growing rabbits. FO = Flaxseed oil; FOA = Flaxseed oil + Allicin; FOL = Flaxseed oil + Lycopene; FOP = Flaxseed oil + Punicalagin. ^a–c^ Different superscript lowercase letters in the same column color indicate significant differences (*P* < 0.05)

The antioxidant profile (Fig. 2) of heat-stressed rabbits fed diets enriched with FSO improved significantly by treating with ALC, LCO, or PCA. Serum MDA significantly (*p* = 0.001) declined with ALC, LCO, or PCA compared to the CON and FO groups. The PCA rabbits had the highest TAC concentration than in the other groups.

The liver and kidney functions in Table 5 showed that FSO significantly (*p* = 0.001) increased serum GOT, GPT and creatinine concentration, while the highest levels of globulin recorded in FOL and FOP rabbits.

**Table 5:** Effects of flaxseed oil with or without allicin, lycopene and Punicalagin on blood liver and kidney function

Treatment	control	Flaxseed oil				SEM*	*P* value
		alone	allicin	lycopene	Punicalagin		
Liver function							
T. protein	5.58	5.78	5.59	5.46	5.61	0.08	0.836
Albumin	4.21	4.03	4.04	3.58	3.57	0.09	0.080
Globulin	1.37^b^	1.75^ab^	1.56^ab^	1.89^a^	2.04^a^	0.07	0.007
GOT	16.80^b^	23.35^a^	15.40^b^	14.88^b^	15.83^b^	0.75	0.0001
GPT	9.10^b^	13.93^a^	8.55^b^	7.63^b^	6.63^b^	0.63	0.0001
**Kidney function**							
Urea	20.15	17.55	13.53	14.43	14.80	0.84	0.056
creatinine	0.25^ab^	0.40^a^	0.14^b^	0.13^b^	0.12^b^	0.03	0.001

Means in row with different superscript letters (a-b) are significantly different (*p* ≤ 0.05), *SEM = Stander Error Mean

### The muscle fat acids analysis (ω3 & ω6), cholesterol and immune response

**Fig. 3:**
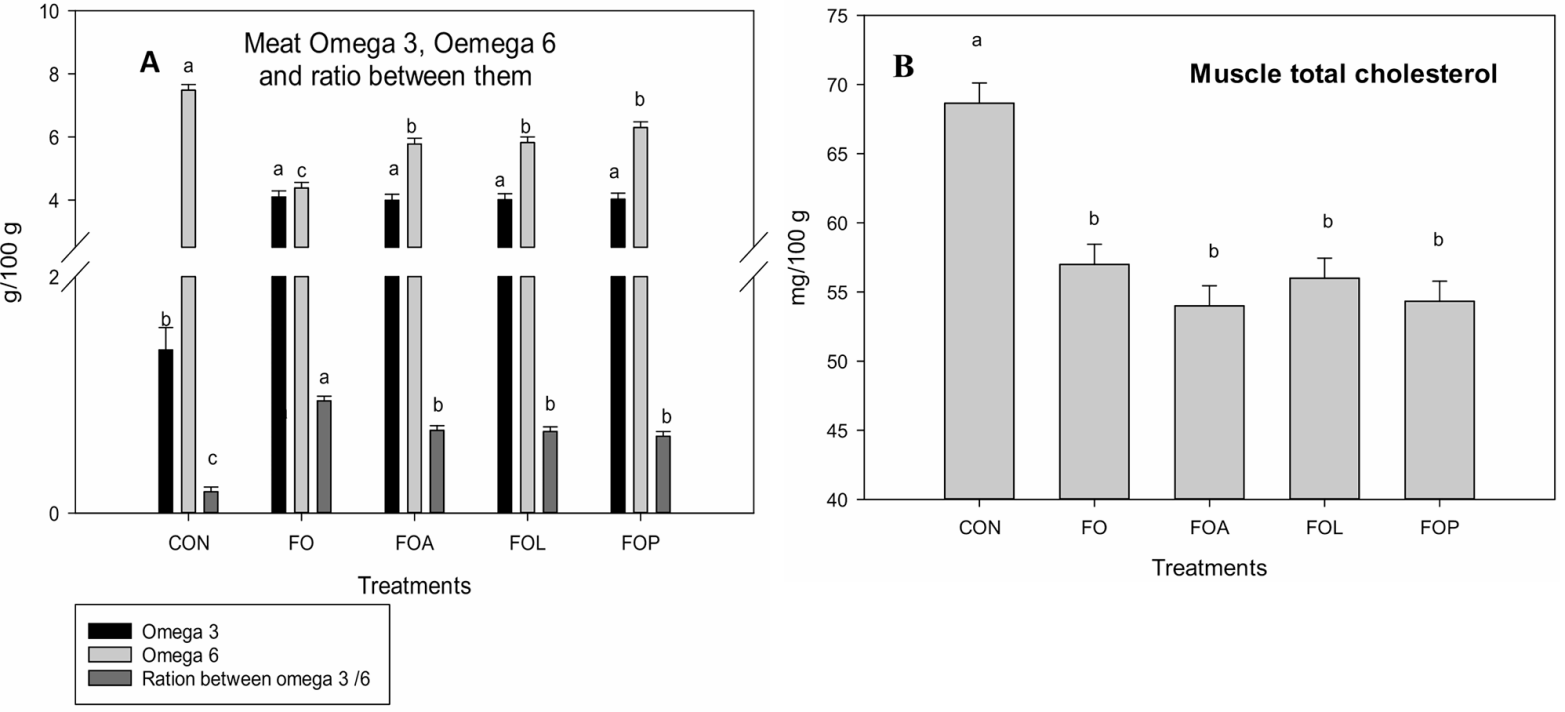
Effects of Flaxseed oil with or without allicin, lycopene and Punicalagin on meat omega 3; omega 6 and ratio between them (**a**) and meat T. cholesterol (**b**) of heat stressed V-line growing rabbits. FO = Flaxseed oil; FOA = Flaxseed oil + Allicin; FOL = Flaxseed oil + Lycopene; FOP = Flaxseed oil + Punicalagin. ^a–c^ Different superscript lowercase letters in the same color column indicate significant differences (*P* < 0.05)

**Fig. 4:**
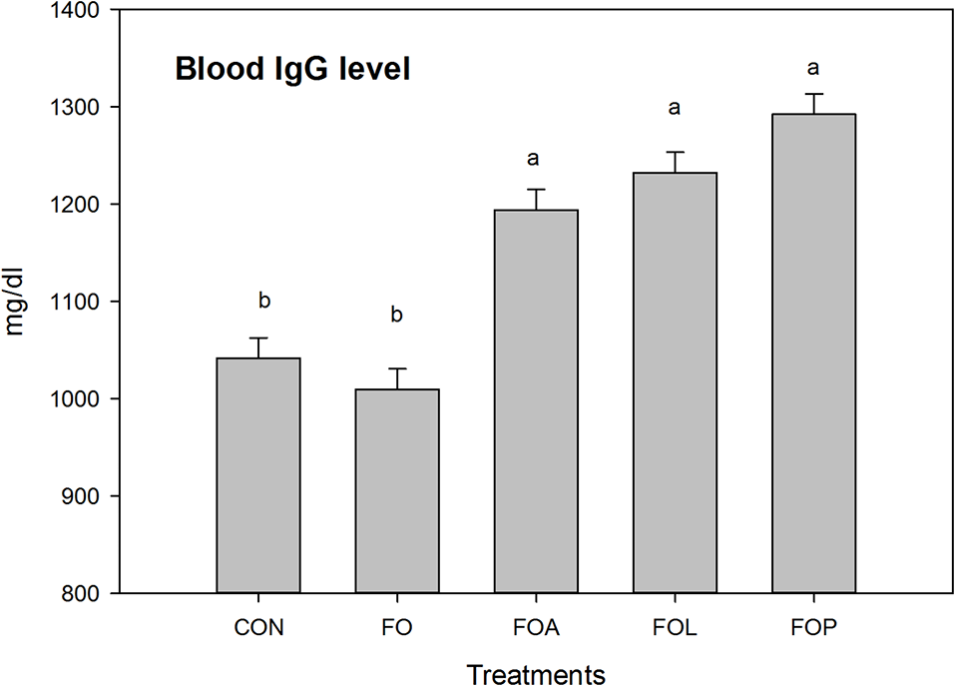
Effects of Flaxseed oil with or without allicin, lycopene and Punicalagin on blood IgG value of heat stressed V-line growing rabbits. FO = Flaxseed oil; FOA = Flaxseed oil + Allicin; FOL = Flaxseed oil + Lycopene; FOP = Flaxseed oil + Punicalagin. ^a–b^ Different superscript lowercase letters indicate significant differences (*P* < 0.05)

The data described in Fig. 3a indicated that the muscle fat acids analysis ω3, ω6 and their ratio were significantly affected by experimental treatments. All flaxseed oil with or without ALC, LCO, or PCA significantly (*p* = 0.001) rise ω3 compared to CON group. Although FO rabbits significantly recorded the maximum value of ω6 compared to other groups. The best ratio between ω3 and ω6 was recorded in FO groups whereas CON group recorded the lowest value. The results of Fig. 3b revealed that all treatments with flaxseed oil alone or with ALC, LCO or PCA significantly decreased the values of meat T. cholesterol compared to CON. Serum IgG concentration (Fig. 4) significantly (*p* = 0.001) rises with the antioxidant substance used in this study compared to the CON and FO groups.

## Discussion

Rabbits are particularly susceptible to heat stress, as they have a thick coat of fur and lack sweat glands to help regulate their body temperature. When exposed to high temperatures, rabbits can experience a range of negative effects, such as decreased growth rate and meat quality [28], low nutrient absorption and metabolism, and reduced immunity [29], plus heat stress can affect muscle composition, resulting in tougher and less palatable.

In this study, the THI values throughout the experimental period appeared to show that the rabbits suffered moderate to severe heat stress, as shown in Table 2. The result of the physiological parameters revealed that all treatments do not affect the thermoregulation indices (RR and RT data) of the growing rabbit suffering from heat stress, due to a short experiment period that is not enough to affect the physiological parameters. The current results are not in agreement with results reported by [30] who described that fat supplementation (such as FSO) increased physiological parameter responses. While El-Diahy [31] found that RT and RR in heat-stressed cows during the summer period decreased significantly with FSO treatment compared to the CON group. This difference in previous works can clarify by different experimental duration, the age, and species of animals.

There was a non-significant variation in feed conversion due to the different treatments. Although the feed intake decreased significantly in FO group which in turn, results decreased both FBW and BWG while growth performance improved when mixed FSO with the ALC, LCO, or PCA groups. The enhancement in growth performance could be due to the useful effects of ALC, LCO, or PCA as a natural antioxidant substance which protects nutrients of meat from oxidation. The decline in feed intake and BWG by fed rabbits diet contain FSO only get al.ong with [5, 32, 33] findings. Unlike the study findings, Fawzy and Nagy [34] reported that no pronounced differences in BWG were found between CON and FSO rats.

The decrease in feed intake and FBW of FSO rabbits could be attributed to altered food taste and palatability, reduced mineral availability such as calcium, iron, magnesium, and zinc due to phytic acid, or impaired protein digestion caused by interactions with amino acids and enzyme inhibition [35].

Furthermore, Moghaddam [36] administered that using punicalagin up to 2 mg/kg/day for the month increased the body weight of adult mice. Likewise, Abdel-Rahman [37] noted that the decrease in FBW induced by bisphenol A was slightly enhanced by lycopene treatment. Our result disagreed particularly with those of Saleh [5], who revealed that FSO has a nonsignificant effect on FBW and BWG of male growing New Zealand white rabbits, but the feed conversion ratio was improved when FSO was mixed with an antioxidant agent such as organic selenium. The improvement in the growth parameters of heat-stressed rabbits that were noticed in response to natural antioxidant supplementation could be due to their protective effects and stimulation of digestive enzymes [38]. Additionally, Allicin enhances intestinal flora and increases the count of cecum anaerobic bacteria with inhibited bacteria growth (pathogenic and non-pathogenic strains) in the digestive tract [29].

The results agreed with Farag [39] founded that a supplemented diet with 100, 200, and 300 mg / kg of LCO increased FBW and BWG in chicken production [40]. also observed an improvement in daily BWG in rabbits fed diets containing 100 or 200 mg of LCO / kg of diet. The known antioxidant properties of lycopene could improve immune function and combat oxidative stress, potentially leading to better health and growth [41].

On the contrary, studies on ALC treatment did not show significant effects on body weight. The usage of garlic powder as a source of ALC to broiler diets did not alter body weight or weight gain [42]. Similarly [43], stated that there were no changes in weight gain in chickens fed garlic at different doses. Furthermore [44], found that rabbit fed heat stressed rabbit ALC and LCO had the best FBW, BWG, and feed conversion ratio compared to CON due to reduction in liver TNF- α levels. ALC, LCO, or PCA have a favorable effect in improving rabbit performance, in this context, when the farm maintains standards of hygiene. FSO diet supplementation increased serum T. lipid, cholesterol, triglycerides, and v-LDL. The allicin diet worked to alternate and get down the levels of blood triglyceride and v-LDL of rabbits growing flaxseed oil. Furthermore, LCO has a helpful effect on reducing the blood cholesterol concentration of FO-growing rabbits. Additionally, each of ALC, LCO, or PCA as an active component significantly decreased the serum total lipid of the FO group.

Saleh [5] reported that FSO supplementation with or without antioxidant substance (organic selenium) reduced total blood cholesterol and low-density lipoprotein, while blood HDL increased in FSO with antioxidant substance. The decrease in cholesterol in FOL treatment may be due to modulation of cholesterol metabolism or blocking cholesterol biosynthesis [45]. Furthermore, LCO or raw tomatoes prevent and reduce LDL oxidation, increase HDL level of HDL, and improves the serum lipid profile [46–48] due to its ability to suppress HMG-CoA reductase activity, improve LDL receptor function in macrophages, and effectively neutralise free radicals [49].

Additionally, LCO decreases blood total lipids and cholesterol, oxidized LDL and interleukin-1, and improves total antioxidant capacity [48]. Adding LCO to a high-fat diet significantly decreases serum total cholesterol and improves serum HDL [50].

Reduced blood triglycerides in rabbit-fed diets, including FSO and ALC, by decreasing the activity of specific liver enzymes, including fatty acid synthase, glucose-6-phosphate dehydrogenase and HMG-CoA reductase, which are responsible for lipids and cholesterol [51]. Allicins have an excellent antioxidant property that reduces oxidative stress, which contributes to hypertriglyceridemia. Allicin reduces oxidative stress and inflammation in fatty liver disease mice by decreasing obesity, liver enzymes and the expression of microsomal protein cytochrome [52]. Overall, all ALC, LCO or PCA as an antioxidant active component reduced fatty liver disease through optimized lipid metabolism.

Rabbits exposed to high summer temperatures experienced a significant reduce in serum total antioxidant capacity and an increase in serum MDA levels [53, 54]. Lipid peroxidation, a hallmark of cellular stress, is a self-sustaining chain reaction that progressively damages cell membranes through oxidative processes. Heat stress with lipids improves MDA, a major end of lipid peroxidation waste product [55]. Therefore, including FSO with various feed additives as used in this study appeared to provoke the harmful effects of heat stress (Fig. 2). The TAC increased, while serum MDA decreased in rabbits treated with FOA, FOL, and FOP, indicating a reduction in oxidative damage compared to the CON group.

The enhancement of antioxidant profile of FSO with the ALC, LCO, and PCA groups indicates a reduction in oxidative damage, which may be the result of a dropping in the pro-oxidant/antioxidant balance (redox balance), leading to the protection of DNA, lipids, and proteins, as well as the regulation of the cell cycle [56]. Besides, LCO effectively neutralizes singlet oxygen (^1^O_2_) and peroxyl radicals (LOO•) [57]. The conjugated double bond system of lycopene forms a highly efficient system of delocalised electrons along its polyene chain. This unique structure allows lycopene to readily interact with and neutralize unstable molecules containing unpaired electrons, known as free radicals [48]. As previously reported by [58], LCO exhibits a greater capacity than β-carotene to neutralize singlet oxygen, capture peroxyl radicals, and suppress lipid peroxidation.

Additionally, ALC scavenging free radicals, neutralising harmful molecules that damage cells by activating antioxidant enzymes, including SOD, catalase, and glutathione peroxidase, which further detoxify harmful substances or stimulate the production of endogenous antioxidants such as glutathione, enhancing natural defence system of animal [40].

Regareeing PCA, it possesses numerous hydroxyl groups, allowing it to scavenge free radicals directly, reducing oxidative stress in the blood, plus, PCA gets metabolized into ellagic acid, another potent antioxidant with free radical scavenging abilities and can stimulate antioxidant enzyme activity as a further enhancing the body’s natural defences [17].

Protein profiles and liver enzyme activities are key indicators of hepatic health, particularly for detecting liver cell damage (cytolysis). These enzymes are sensitive to toxic substances and their levels can reveal the severity of liver damage [59]. Besides, heat stress can damage hepatocytes, leading to elevated liver enzymes (GOT and GPT) that found on cell membrane surface and increased blood triglycerides (TG). Antioxidants such as lycopene and allicin can improve rabbit liver health under oxidative stress [44], but its mechanism remain unclear.

Regulation of cytoplasmic glutathione levels protects cells from oxidative stress [60]. All flaxseed oil treatments significantly increased blood globulin and decreased GOT and GPT level compared to CON group, that result is getting along with [1]. The results suggest that ALC, LCO or PCA can improve liver function and reduce liver enzyme activity in heat stressed rabbits. For kidney function, blood urea levels not affected by all experimental treatments, while creatinine level significantly decreased by supplementation with ALC, LCO, or PCA in FO rabbits. These results may be due to the reduction of oxidative stress and inflammation, contributing to improved kidney function of heat-stressed rabbits.

To enhance the α-linolenic acid in heat-stressed rabbit meat, we propose adding FSO during the last three weeks of the fattening period. Our findings indicate that FSO, potentially combined with ALC, LCO, or PCA, can increase ω-3 in rabbit meat. This dietary intervention could provide consumers with a healthier and more functional meat product. Importantly, altering the lipid composition of animal feed directly influences the nutritional value of the meat consumed by humans [61].

Rabbits fed a diet supplemented with PUFA-rich oils, like FSO, exhibit increased polyunsaturated/saturated fatty acid ratios, higher levels of α-linolenic and linoleic acids, and a favorable ωn-3/ωn-6 ratio in their muscle tissue. Furthermore, palmitic acid levels decrease [62, 63]. Our results corroborate previous studies that have observed significant elevations in total ω3 (PUFA) concentrations, coupled with reductions in saturated fatty acids (SFA), in monogastric animals supplemented with flaxseed [64–66], while others did not notice much difference [67–69].

Furthermore [70], reported that incorporating 8% or 16% flaxseed into rabbit diets reduced saturated and monounsaturated fatty acids by 22% and 24%, respectively, while increasing PUFA by 36%.

Utilizing natural antioxidants with FSO is a good strategy to improve the meat content of PUFA by preventing lipid radical-induced damage to cell membranes [48,71]. demonstrated that incorporating 10% extruded flaxseed in pork diets elevated levels of linoleic acid, linolenic acid, total PUFAs, and ω3 and ω6 PUFAs, while decreasing saturated fats. Furthermore [66], found that including 3% flaxseed in rabbit feed effectively enriched meat with alpha-linolenic acid without compromising product quality.

The meat cholesterol of growing rabbits suffering from heat stress fed a diet supplemented with FSO with or without ALC, LCO or PCA significantly decreased. In agreement with the results of my study, a decrease in the cholesterol content of leg meat was observed in chickens fed diet supplemented with lycopene [45].

As shown in Fig. 3 IgG significantly increased by dietary supplementation of FSO with ALC, LCO or PCA in the present study. These results suggested that these substance with FSO has a potential synergistic effect on immune responses, aligning with the observed antioxidant properties (Fig. 2). Also antibody titers increased following antioxidant supplementation [72].

Dietary antioxidants may help to promote formation and maintenance of healthy immune cells by Neutralizing harmful free radicals to safeguard cellular health [73]. My findings supported by the good health status of rabbits. The immunomodulatory properties of each FSO with each of ALC, LCO or PCA could work together to boost IgG levels and overall immune function of heat stressed rabbit.

While flaxseed supplementation, with or without additional antioxidants, significantly reduced muscle total cholesterol, the work of [74] suggests that a balanced intake of essential unsaturated fatty acids can modulate cholesterol levels by influencing hepatic HMG-CoA reductase activity. Additionally [63], observed elevated levels of linoleic and α-linolenic acids in rabbits fed a flaxseed oil diet, leading to a significant reduction in the ω6/ω-3 PUFA ratio. More research warranted to investigate the specific mechanisms by which this combination influences various physiological parameters such as gut microbiota modulation and study how these changes improve performance and health and of heat stress rabbits with study any changes in gene expression related to stress response, antioxidant defence.

## Conclusions

The data presented here clearly show that the heat stressed growing rabbit diets fed FSO with ALC, LCO or PCA for the last 3 weeks before slaughter consider a good strategy to improve meat quality by enriched it with ω3 and reduce ω6, ratio between ω3 and 6 and cholesterol level with a significant improvement in immunity status (IgG levels). In addition, the use of flax seed oil with lycopene successfully enhances growth performance, besides using allicin or lycopene with FSO improved the lipid and protein profile. Punicalagin with FSO, the best antioxidant status of heat stressed rabbits found in PCA treatment.

## Data Availability

The data used to confirm our study’s findings are included in the article, and data coding is available from the corresponding author upon reasonable request.

## Abbreviations

AlC: Allicin
ANOVA: Analysis of variance analyzed
CON: Control
FSO: Flaxseed oil
GGT: Gamma-glutamyl transferase
GLC: Gas chromatography
GOT: Glutamate oxaloacetate transaminase
GPT: Glutamate pyruvate transaminase
IgG: Immunoglobulin G
LCO: Lycopene
LDL: Low density lipoprotein
MDA: Malondialdehyde
ω3: Omega 3
ω6: Omega 6
PCA: Punicalagin
PUFA: Polyunsaturated fatty acids
RH: The relative humidity percentage
RR: Respiration rate
RT: Rectal temperature
SFA: Saturated fatty acids
SOD: Superoxide dismutase
T. lipid: Total Lipid
TAC: Total antioxidant capacity
THI: Temperature humidity index
v-LDL: Very low-density lipoprotein
